# Comparison of the Genital Microbiomes of Pregnant Aboriginal and Non-aboriginal Women

**DOI:** 10.3389/fcimb.2020.523764

**Published:** 2020-10-29

**Authors:** Nicole K. Dinsdale, Natalia Castaño-Rodríguez, Julie A. Quinlivan, George L. Mendz

**Affiliations:** ^1^School of Medicine, Perth, The University of Western Australia, Perth, WA, Australia; ^2^School of Biotechnology and Biomolecular Sciences, The University of New South Wales, Kensington, NSW, Australia; ^3^Institute for Health Research, The University of Notre Dame Australia, Fremantle, WA, Australia; ^4^School of Medicine, Sydney, The University of Notre Dame Australia, Darlinghurst, NSW, Australia

**Keywords:** Australian aboriginal women, pregnancy, vaginal microbiome, placental microbiome, infection, preterm birth, neonatal sepsis

## Abstract

The genital microbiomes of women varies with racial background. Preterm birth and early-onset neonatal sepsis are two outcomes associated with genital infections during pregnancy. The rate of preterm birth in Aboriginal Australian mothers is high, as is the rate of early-onset sepsis in their infants. To date, no studies have been conducted to investigate genital microbiome taxa associated infection in this group of women. A prospective cohort study to characterize the vaginal and placental microbiomes of a group of these women from the Pilbara region was conducted at the Hedland Health Campus in Western Australia. Included in the study were gravidae Aboriginal (*n* = 23) and Non-aboriginal (*n* = 27) women in labor or for planned lower uterine segment Caesarean section. Employing sterile swabs, vaginal samples were obtained under sterile conditions immediately prior to vaginal delivery or planned Caesarean section; and placental samples were obtained under the same conditions during labor. Taxa present in the samples were identified by 16S rRNA amplicon sequencing (V4 region, 515F-806R). Taxon identity and abundance were established from Operational Taxonomic Unit (OTU) counts. Statistical analyses combining clinical metadata and sequencing results were employed to determine associations of taxa with racial background. The findings of this work served to enhance the current understanding of microbiota associated with health and disease in Aboriginal and Non-Aboriginal women. Differences were found between the vaginal and placental microbiomes of Aboriginal and Non-aboriginal women during pregnancy, as well as notable differences between the abundance of specific taxa in each racial group. The relative abundances of specific taxa were significantly different between participants with clinical signs of infection and those with healthy pregnancies. This work will contribute to understanding the causes of differences in rates of infection-driven preterm birth in various racial populations.

## Introduction

Genital bacterial community profiles differ with racial background. Dominant *Lactobacillus* in the vaginal flora of pregnant women is more prevalent amongst Caucasian and Asian women, and anaerobic bacterial communities are more common amongst African American and Hispanic women (Ma et al., 2012; Romero et al., 2014; MacIntyre et al., 2015; Freitas et al., 2017; Fettweis et al., 2019). Studies of the vaginal microbiome also have found racial differences in the context of preterm birth (Callahan et al., 2017; Elovitz et al., 2019). The associations of intrauterine microbiota with racial background have been studied for placental samples of African, Caucasian and Chinese women (Zheng et al., 2015; Collado et al., 2016; Doyle et al., 2017; Gomez-Arango et al., 2017; Parnell et al., 2017; Zhou et al., 2018), but there are little data on the influence of racial background on other intra-amniotic bacterial populations. There have not been investigations of the genital microbiota of pregnant Australian Aboriginal women; this study aims at addressing this gap by providing data on their vaginal and placental microbiomes and comparing them to the genital microbiota of Non-aboriginal women from the same Pilbara region in Western Australia. Knowledge of bacterial taxa residing in the vagina and placenta in normal pregnancies provides a foundation for healthcare professionals to identify abnormal flora, enabling the possibility of targeted prevention of genital infections.

The genital microbiomes have an important role in maternal and neonatal health. At the beginning of pregnancy, microbial richness and diversity of bacterial populations are reduced. At the same time, in healthy pregnancies the prevalence of potential pathogens is reduced too (Goltsman et al., 2018). The taxonomic composition of the microbial community of the vagina remains stable during pregnancy with an increase of the microbial diversity before birth of a healthy infant at term (DiGiulio et al., 2015). The historical view that the amniotic cavity constitutes a sterile environment has been challenged by findings that the healthy maternofetal unit is colonized with microbes, and that this is a prerequisite for immune maturation as well as metabolic and hormonal homeostasis (Staude et al., 2018). However, a review of recent findings concluded that the evidence supporting the hypothesis of a uterine microbiome is extremely weak (Perez-Muñoz et al., 2017). In addition, a recent study with placental samples concluded that the human placenta does not have a microbiome (De Goffau et al., 2019). Nonetheless, there is evidence that the placenta, the amnion, and the fetus share large proportions of a common microbiome, and that the maternal microbiome drives the development of the fetal immune system (Collado et al., 2016; De Aguero et al., 2016) These data lead to the conclusion that the composition of the vaginal microbiota is tightly regulated during pregnancy and that the switch to the non-pregnant situation precedes and maybe even triggers birth (Romero et al., 2014; DiGiulio et al., 2015; Freitas et al., 2017).

Owing to the polymicrobial nature of the genital microbiota the definition of what is normal or abnormal genital tract microflora is very difficult; normal microflora is assumed to be present in the absence of disease (Lamont, 2015). Spontaneous preterm labor leading to preterm birth (PTB) is recognized as a syndrome caused by a number of pathological processes leading to activation of the common terminal pathway of parturition. Abundant evidence supports the view that local or systemic infection or inflammation is a major cause of early PTB (Lamont, 2015). Bacterial infections threaten pregnant women and the fetus by gaining access to gestational tissues, such as the decidua, placenta, and fetal membranes (Vinturache et al., 2016). There are correlations between the risk of PTB and genital bacterial populations in both the vaginal (Wen et al., 2014) and intrauterine (Mendz et al., 2013) microbiota. These correlations differ between racial groups; for example, a study observed significant associations of vaginal bacteria and PTB in African and Hispanic American women, but Caucasian Americans did not show significant associations between vaginal microbiota and birth outcome (Wen et al., 2014).

It is estimated that 25%-40% of PTB are attributable to intrauterine infection (Goldenberg et al., 2008), and the relationship between the presence of pathogens and spontaneous preterm birth is complex. These findings underline the need to consider women's race when evaluating the links of specific genital bacteria with preterm birth. Similarly, early-onset neonatal sepsis (EOS) is associated with pathogenic microorganism acquired from the mother during pregnancy or at birth (Schrag et al., 2016). Firm connections have been established between several taxa such as *E. coli* and *Streptococcus* B and EOS, but the association of other taxa such as *Enterococcus* and *Haemophilus* with this type of sepsis appears to depend on human populations (Simonsen et al., 2014; Singh et al., 2019).

The rate of preterm birth (PTB) of Aboriginal Australian mothers is 14% and of Non-aboriginal mothers is 8% (Australian Institute of Health and Welfare, 2016), the former is higher than those of many underdeveloped countries. Spontaneous preterm birth was the most commonly identified cause of perinatal death in infants of Aboriginal and/or Torres Strait Islander (ATSI) mothers: 26% compared with 19% for Non-indigenous mothers (Australian Institute of Health and Welfare, 2018). Historically, infants of ATSI mothers have been over-represented in EOS data, although recent studies suggest that this rate may be decreasing (Braye et al., 2019). The current study investigated the presence of bacterial taxa associated with clinical signs of infection and PTB or EOS in the participant Australian Aboriginal women with the aim of providing data that would serve to devise strategies to reduce adverse pregnancy outcomes in this population.

## Methods

### Study Design and Participants

A prospective cohort study was performed at Hedland Health Campus (HHC), located in Port Hedland, Pilbara region, Western Australia. Aboriginal people account for 16.2% of the Pilbara's population. Inclusion criteria admitted pregnant women with no known fetal anomalies, 18 years of age or older, and ability to provide informed written consent. Racial background was determined by self-identity as Aboriginal or non-Aboriginal Australian. Included in the study were pregnant Aboriginal (*n* = 23) and non-Aboriginal (*n* = 27) women recruited upon presenting to the birth suite in labor or for planned lower uterine segment Caesarean section (LUSCS). Demographic data were collected from maternal and infant medical records and de-identified. It included age, gravidity, parity, gestational age at birth, infection during pregnancy, and information on previous pregnancies. Vaginal and placental swabs from each woman were obtained in theater prior to vaginal delivery or planned Caesarean section and during labor, respectively.

### Ethics Approval

Participants volunteered to be in the study and provided written informed consent. The study was undertaken following approvals from WA Country Health Service Human Research Ethics Committee (HREC #2015/40) and Western Australian Aboriginal Health Ethics Committee (HREC #686).

### Study Protocol

Previous investigations found that during pregnancy bacterial communities are stable in the introitus, midpoint and posterior fornix of the vagina (Consortium HMP, 2012; Huang et al., 2015). In this study, vaginal samples were collected from the vaginal midpoint (3 cm from introitus) using a sterile plastic swab with a Dacron tip. Vaginal samples were collected under sterile conditions by a qualified midwife on arrival to birth suite for women in spontaneous labor, or in theater immediately prior to surgery in women booked for LUSCS. Placentas were collected under sterile conditions during labor and stored at 4°C; placental samples were obtained by swabbing between the amnion and chorion layers of the placenta using the same type of sterile plastic swab. This method reduced sampling potential microbial contaminants from the vagina during the passage of the placenta through it. Two samples were collected from the same placental site for each participant for quality assurance purposes. Swabs were obtained from the vagina and the placenta of each participant, labeled for their participant origin but de-identified from the participant, and immediately preserved frozen at 0°C in sterile tubes until DNA extraction.

### DNA Extraction, Amplification, and Sequencing

DNA extraction and purification were performed using the QIAamp DNA Mini Kit according to the manufacturer's instructions (QIAGEN, Chadstone Center, VIC, Australia); the concentration and quality of the DNA was assessed using a Nanodrop ND-1000 Spectrophotometer (Nanodrop Technologies; Wilmington, DE, USA). To minimize environmental contamination, DNA was extracted following a recommend practice (Eisenhofer et al., 2019).

The composition of the microbial communities was determined at the Ramaciotti Centre for Genomics (University of NSW, Sydney, Australia) by amplicon sequencing using an Illumina MiSeq instrument (2 × 250 bp chemistry). The 16S rRNA gene was amplified using the KAPA HiFi HotStart ReadyMix (95°C for 3 min, 25 cycles of 95°C for 30 s, 55°C for 30 s, 72°C for 30 s, followed by a final step of 72°C for 5 min) and the Earth Microbiome primers (515F-806R). Indices and Illumina sequencing adapters were attached using the Nextera XT Index Kit according the manufacturer's instructions. The Ramaciotti Centre is an institution with a quality management system according to the ISO/IEC17025:2017 Standard, and it follows recommended best practice protocols (Eisenhofer et al., 2019).

All placental and vaginal samples were analyzed individually and keeping track of their paired origin. Raw reads were quality checked, trimmed and aligned using the MiSeq standard operating procedures implemented in Mothur v1.39.1 with the SILVA SEED 16S rRNA reference database and classification with RDP (read depth 81,681 ± 7,145 clean reads/per sample). DNA extraction procedures and microbiome analyses have been previously utilized and validated.

Reagents in the QIAGEN extraction kit were included in the analyses as negative controls. To address the potential contamination of the placental microbiome by taxa present only in the vagina, all placental and vaginal paired samples were analyzed individually, and the taxa present as well as their relative abundances were compared. The data provided evidence that a possible contamination of the placental microbiome by the vaginal microbiota at the time of delivery was negligible (Supplementary Table 1). All OTU with an abundance >1% in the negative controls (*n* = 21) were excluded from the analyses. Application of these quality control processes left a total of 1,667 OTU. A hierarchical cluster was further generated and consistency was found to be optimal between biological replicates.

### Data Analyses

The de-identified demographic data collected from the clinical records of mother and baby were analyzed using the Statistical Package for the Social Sciences (SPSS), Version 2.2. One-way Analyses of Variance (ANOVA) were carried out to determine differences in the microbiomes of Aboriginal and Non-Aboriginal cohorts. Logistic regression modeling was conducted to control for potential confounding variables, such as previous PTB history, maternal age and drug and alcohol use during pregnancy.

Taxon relative abundance was determined from sequence counts and expressed in percent. Genital taxa sequence data were analyzed to determine species diversity and richness, and Shannon's Diversity Index for each participant and the entire cohort. To evaluate beta-diversity in this cohort, multivariate analyses including Distance Linear Model (distLM) analyses, non-metric multidimensional scaling (nMDS) and PERMDISP were performed on Bray-Curtis resemblance matrix of square-root transformed relative abundances using Primer-E v7 (Clarke, 1993).

Probability tests were used to combine the clinical metadata and sequencing data to generate *p*-values of the association of microbial taxa with genital infection. Multivariate Association with Linear Models analyses (MaAsLIN) were conducted to evaluate associations between clinical metadata and microbial community abundance. After removing confounders, Linear Discriminant Effect Size analyses (LEfSe) (Segata et al., 2011) were performed to compare taxa enrichment between vagina and placenta genital locations and Aboriginal and Non-aboriginal women. These analyses were conducted using the Galaxy web application (Afgan et al., 2018). Taxon prevalence was calculated as the number of samples with a relative abundance > 1% expressed as a percentage of the total number samples. To focus on taxa with minimum frequencies in the populations rather than on those whose presence is circumscribed to a few individuals, the frequency with which taxa were found in the samples were included in the analyses; only taxa with frequencies equal or higher than 15% were reported.

## Results

### Clinical Characteristics of the Study Population

Participants were grouped in two self-identified racial categories almost equally represented: Aboriginal (*n* = 23) and Non-Aboriginal (*n* = 27). The racial composition of the Non-aboriginal participants was 76% Caucasian, 16% Asian, 8% Middle East. Demographic, clinical and pregnancy outcome characteristics data of the two groups of women are summarized in Table 1. The maternal age was lower for the Aboriginal participants (25.6 years, range 18–34 y.o.) than for the Non-aboriginal participants (29.5 years, range 21–36 y.o.), with a significant difference between them on one-way ANOVA testing (*p* = 0.01). The mean gravidity and parity were significantly higher for the Aboriginal women (G3.1, P2.5), compared to the Non-aboriginal women (G1.8, P1.6).

**Table 1:** Demographic and clinical characteristics of Aboriginal (*n* = 23) and Non-aboriginal (*n* = 27) women.

**Characteristics**	**Aboriginal**	**Non-aboriginal**	***p*-values**
**Mothers**			
Mean age	25.6 years	29.5 years	0.001
Mean gestational age	38.2 weeks	39.1 weeks	0.061
Mean gravidity	3.1	1.8	0.002
Mean parity	2.5	1.6	0.007
Preterm birth history	8 (34.8%)	0 (0%)	0.001
Alcohol consumption	8 (34.8%)	2 (7.4%)	0.015
Smoking consumption	14 (60.9%)	1 (3.7%)	0.000
Illicit drug use	8 (34.8%)	1 (3.7%)	0.004
**Complications during pregnancy**			
Infection	12 (52.2%)	4 (14.8%)	0.004
IUGR	4 (17.4%)	5 (18.5%)	0.920
Hypertension	4 (17.4%)	4 (14.8%)	0.809
Obesity	4 (17.4%)	4 (14.8%)	0.809
APH	2 (8.7%)	4 (14.8%)	0.517
Diabetes	3 (13%)	2 (7.4%)	0.518
None	9 (39.1%)	10 (37%)	0.882
**GBS status**			
Positive	9 (39.1%)	5 (18.5%)	0.369
Negative	7 (30.4%)	14 (51.9%)	0.023
Unknown	7 (30.4%)	8 (29.6%)	–
**Birth mode**			
SVD	17 (73.9%)	12 (44.4%)	–
AVD	1 (4.3%)	1 (3.7%)	–
Elective LUSC	3 (13%)	6 (22.2%)	–
Non-elective LUSC	2 (8.7%)	8 (29.6%)	–
**Problems during labor**			
Maternal fever	1 (4.3%)	3 (11.1%)	0.053
Mean blood loss	569.6 ml	440.7 ml	–
**Neonates**			
Preterm	6 (26.1%)	2 (7.4%)	–
Neonatal sepsis	4 (17.4%)	4 (14.8%)	–

^*^Data are presented as frequencies (proportion percentage in each cohort).

None of the Non-aboriginal participants had a previous history of PTB, and eight Aboriginal women had a background of PTB. Antenatal smoking, alcohol consumption and illicit drug use were significantly higher in Aboriginal women. Antenatal urinary, respiratory, or sexually transmitted infections were found in 52.2% of Aboriginal and 14.8% of Non-Aboriginal participants. Apart from a history of antenatal infection, there were no significant differences in maternal complications at delivery between both groups of participants, only the PTB rate was higher in the group of Aboriginal women (Table 1). Neonatal sepsis was diagnosed by attending clinicians blinded to the study aims and outcomes using established clinical and microbiological criteria (Gerdes, 1991).

### Composition and Diversity of the Genital Microbiomes

Similar OTU, species richness, evenness, and Shannon diversity index were found in the microbiomes of Aboriginal and Non-aboriginal women participating in the study (Table 2). Placental samples, however, showed differences in their OTU and species richness between racial groups in the presence of infection (data not shown). The OTU, richness, and evenness were significantly different between placental and vaginal samples, but not Shannon diversity, and these differences remained after stratifying by racial background (Table 2).

**Table 2:** Number of OTU (S), species richness (d), evenness (J'), and Shannon diversity index (H'), and comparisons of population subgroups stratified by racial background, genital location, and presence of infection.

**Group**	**Average**				***p*****-value**			
	**S**	**d**	**J'**	**H' (log_**e**_)**	**S**	**d**	**J'**	**H' (log_**e**_)**
Aboriginal	36.85	4.06	0.46	1.44				
Non-aboriginal	41.46	4.57	0.44	1.41	0.930	0.940	0.269	0.780
Placental	35.36	3.89	0.46	1.43				
Vaginal	59.17	6.57	0.36	1.34	1.56 E-15	2.13 E-15	3.09 E-09	0.698
Placental (A)	35.72	3.93	0.46	1.43				
Vaginal (A)	58.78	6.53	0.36	1.34	1.37 E-08	1.47 E-08	0.003	0.768
Placental (N-a)	39.39	4.34	0.45	1.41				
Vaginal (N-a)	55.21	6.14	0.36	1.31	2.97 E-08	3.49 E-08	2.05 E-7	0.411
Non-infection	37.17	4.09	0.46	1.43				
Infection	34.91	3.84	0.44	1.37	0.153	0.149	0.025	0.003

**Figure 1:**
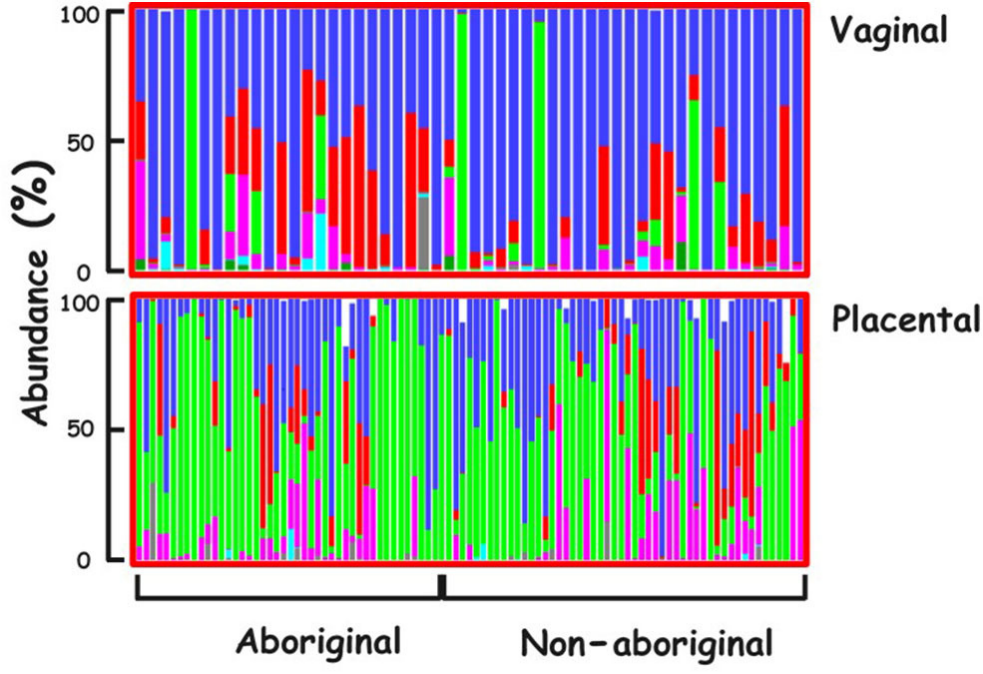
Phyla relative abundances in the vaginal and placental microbiomes of the cohort. Firmicutes (blue), Actinobacteria (red), Proteobacteria (green), Bacteroidetes (magenta), Tenericutes (turquoise), Unclassified (black).

The predominant Phylum in the vaginal microbiomes of all the participant women was Firmicutes, and Proteobacteria in their placental microbiomes. Actinobacteria were more abundant in the vaginal microbiomes of Aboriginal women compared to those of Non-aboriginal women. In the placental microbiomes, Actinobacteria were more abundant in Non-aboriginal than in Aboriginal women (Figure 1).

The compositions of Aboriginal and Non-aboriginal microbiomes appeared similar in non-metric multidimensional scaling 2D and 3D plots using a Bray-Curtis similarity resemblance matrix. However, distLM analyses showed significant differences between Aboriginal and Non-aboriginal microbiomes (*p* = 0.002) (Table 3). Further analyses stratifying by racial background and genital location, yielded an ordination plot with clustered placental samples of each group; but no significant differences were observed in dispersion of the data (*PERMDISP* = 0.52).

**Table 3:** Composition of the microbiomes by racial background (Aboriginal vs. Non-aboriginal), genital location (vagina vs. placenta) and infection (yes vs. no) calculated by a Distance Linear model.

**Variable**	**Pseudo-F**	***p*-value**	**Residual difference**
Racial background	2.842	0.002	147
Genital location	15.646	0.001	146
Infection	0.994	0.435	144

**Figure 2:**
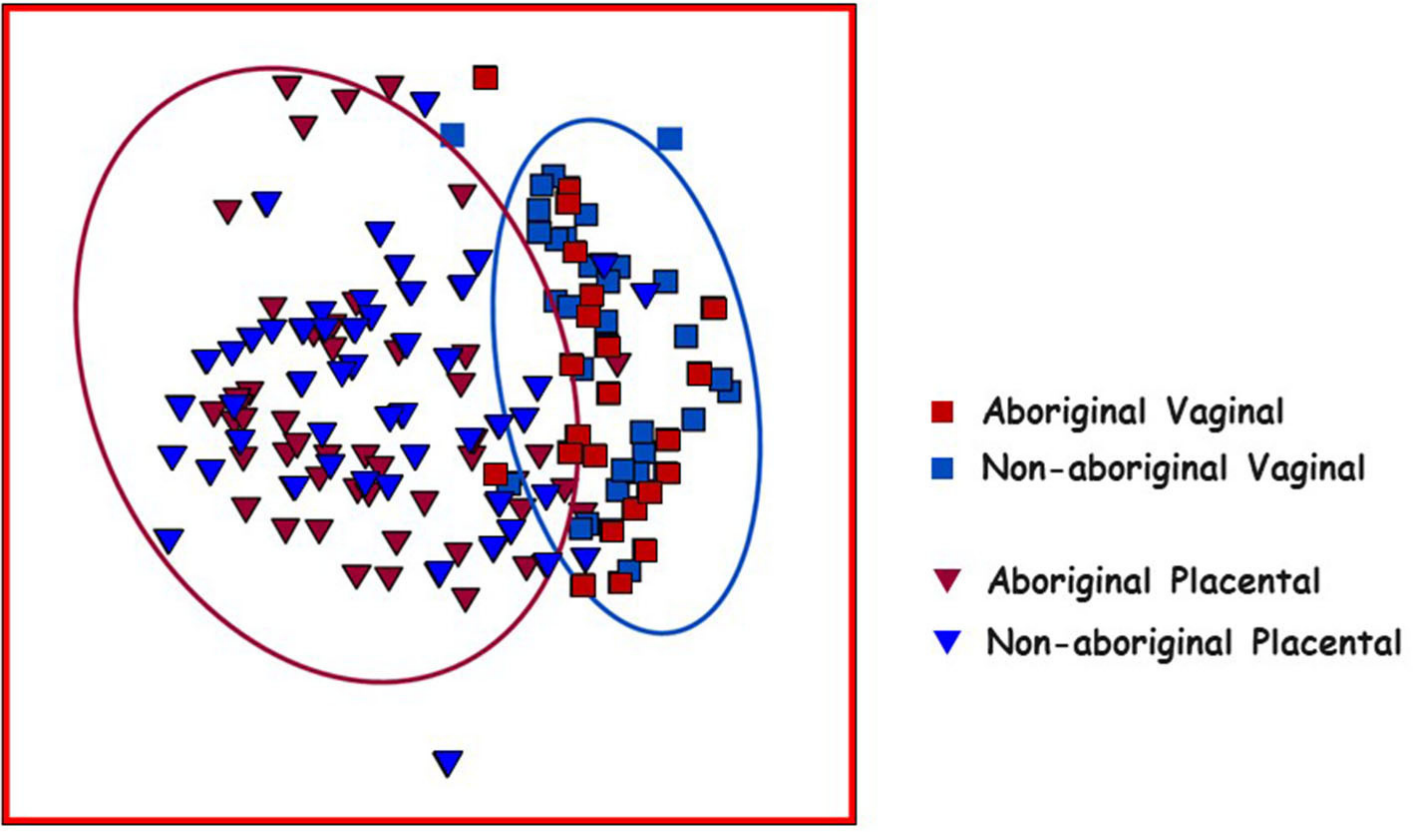
Non-metric multidimensional scaling (nMDS) plot of Bray–Curtis resemblance generated from square root transformed OTU relative abundances. Red squares: Aboriginal participant vaginal microbiomes. Red triangles: Aboriginal participant placental microbiomes. Blue squares: Non-aboriginal participant vaginal microbiomes. Blue triangles: Non-aboriginal participant placental microbiomes. The oval outlines refer to the vaginal (blue oval) and placental (red oval) microbiomes.

Placental and vaginal samples clustered separately (Figure 2), which was confirmed by a significant dispersion between them (*PERMDISP* = 0.05); also, distLM analyses showed significant differences (*p* = 0.001) (Table 3).

### Comparison of Taxa Abundance in Genital Populations and Racial Backgrounds

In both populations of women at the taxon level, LEfSe analyses showed several taxa with enriched relative abundances at each genital location. The genera *Atopobium, Gardnerella, Lactobacillus, Prevotella* and *Staphylococcus* were more abundant in the vagina, and *Enterococcus* and *Flavobacterium* in the placenta (Table 4). In paired vaginal-placental samples of women who gave birth through the vagina, some taxa found in the vaginal microbiomes were present in negligible abundances in the placental microbiomes, and vice versa (Supplementary Table 1). Similar relationships were observed in Caesarean births. These and other instances rule out significant contamination of the placental samples during vaginal delivery. On the other hand, the cases that indicate greater relative abundances in the placental microbiomes suggest access to the placenta via routes other than ascending from the vagina, but do not rule out completely a possible migration of taxa to the amnion from the vagina during pregnancy.

**Table 4:** Differences in the relative abundances between vaginal and placental taxa.

**Taxon**	**Location**	**LDA Score**	***p*-value**
*Atopobium*	Vagina	3.792	<0.001
*Gardenerella*	Vagina	4.314	<0.001
*Lactobacillus crispatus*	Vagina	5.001	0.001
*Lactobacillus iners*	Vagina	4.525	0.003
*Prevotella*	Vagina	3.803	<0.001
*Staphylococcus*	Vagina	3.982	<0.001
*Enterococcus*	Placenta	3.950	0.011
*Flavobacterium*	Placenta	3.939	0.017

Differences in taxa relative abundances were identified between the microbiomes of Aboriginal and Non-aboriginal women. Table 5 shows genera with significantly different relative abundances and frequencies > 15% in each racial background. The taxon with highest relative abundance among the genital taxa associated with Aboriginal participants was *Lactobacillus iners* (OTU0002, 99% similarity), and associated with Non-aboriginal women was the taxon *Lactobacillus crispatus*/*acidophilus/helveticus* (OTU0001, all 99% similarity). These results were confirmed by MaAsLIN analysis (*p* = 2.91 × 10^−4^). The proportion of *Lactobacillus spp*. in vaginal samples followed a bimodal distribution with approximately 30% of samples with < 1% relative abundance, and 30% of samples with relative abundances 60–100%. Other genera found with higher relative abundance associated with Aboriginal women were: *Atopobium, Bifidobacterium, Prevotella, Pseudomonas*, and *Ureaplasma*. Enriched relative abundance genera associated with Non-aboriginal women were *Acinetobacter, Anaerococcus, Streptococcus*, and *Viellonella* (Table 5).

**Table 5:** Taxa with significantly different relative abundances in Aboriginal and Non-aboriginal women.

**Taxon**	**Racial group**	**LDA score**	***p*-value**
*Atopobium*	Aboriginal	3.622	0.012
*Bifidobacterium*	Aboriginal	2.898	<0.001
*Lactobacillus iners*	Aboriginal	4.651	0.001
*Pseudomonas*	Aboriginal	4.480	0.035
*Prevotella*	Aboriginal	3.197	0.025
*Ureaplasma*	Aboriginal	3.174	0.045
*Acinetobacter*	Non-aboriginal	2.918	0.038
*Anaerococcus*	Non-aboriginal	3.573	0.016
*Lactobacillus crispatus*	Non-aboriginal	4.697	<0.001
*Streptococcus*	Non-aboriginal	3.643	0.013
*Veillonella*	Non-aboriginal	3.710	0.010

Significant differences between Aboriginal and Non-aboriginal women were identified by LEfSe analyses in the relative abundances of several genera of vaginal taxa with frequencies > 15% (Table 6). The vaginal communities of Aboriginal women had a greater relative abundance of *Atopobium, Corynebacterium, L. iners, Megasphaera*, and *Ureaplasma*, The vaginal microbiota of Non-aboriginal women had higher relative abundance of the taxa *Anaerococcus, Enterococcus, Escherichia/Shigella, Prevotella, Streptococcus* and *Veillonella* (Table 6).

**Table 6:** Differences in the relative abundances of vaginal taxa between Aboriginal and Non-aboriginal women.

**Taxon**	**Racial group**	**LDA score**	***p*-value**
*Atopobium*	Aboriginal	4.009	0.025
*Corynebacterium*	Aboriginal	3.137	0.009
*Lactobacillus iners*	Aboriginal	4.804	0.007
*Megasphaera*	Aboriginal	3.832	0.006
*Ureaplasma*	Aboriginal	3.281	0.013
*Anaerococcus*	Non-aboriginal	3.455	0.001
*Escherichia/Shigella*	Non-aboriginal	4.319	0.004
*Enterococcus*	Non-aboriginal	4.459	0.026
*Prevotella*	Non-aboriginal	3.318	0.031
*Streptococcus*	Non-aboriginal	3.989	0.017
*Veillonella*	Non-aboriginal	3.885	0.025

Several genera of placental taxa identified by LEfSe analyses and with frequencies > 15% showed significant differences in the relative abundances between Aboriginal and Non-aboriginal women (Table 7). The placental communities of Aboriginal women had greater relative abundances of the taxa *L. iners, Paracoccus*, and *Pseudomonas*. In Non-aboriginal women the taxa *L. crispatus, Streptococcus* and *Veillonella* had greater relative abundances (Table 7).

**Table 7:** Differences in the relative abundances of placental taxa between Aboriginal and Non-aboriginal women.

**Taxon**	**Racial Group**	**LDA Score**	***p*-value**
*Pseudomonas*	Aboriginal	4.742	0.019
*Lactobacillus iners*	Aboriginal	4.632	0.010
*Paracoccus*	Aboriginal	4.185	0.016
*Lactobacillus crispatus*	Non-aboriginal	4.577	<0.001
*Streptococcus*	Non-aboriginal	3.557	0.007
*Veillonella*	Non-aboriginal	3.565	0.037

### Genital Taxa Associated With Infection

The presence of infection did not affect the number of OTU or species richness in the genital microbiomes of both groups of women, but there were significant differences in their evenness and Shannon diversity index (Table 2).

The results of distLM multivariate analyses indicated that the presence of clinical signs of infection did not establish significant differences in the microbiomes of the studied women (*p* = 0.44) (Table 5).

Consistently, infection did not introduce significant differences in dispersion of the data were observed in the vaginal (*PERMDISP* = 0.94) or placental (*PERMDISP* = 0.10) microbiomes of Aboriginal women. Similarly, the presence of infection did not introduce significant differences between the vaginal microbiomes (*PERMDISP* = 0.10) of Non-aboriginal women, but did introduce significant dispersion differences (*PERMDISP* = 0.05) in their placental microbiomes.

Although no significant overall differences were found between the vaginal microbiomes of Aboriginal and Non-aboriginal women and those who experienced an infection, to ascertain whether there were taxa associated with the presence of infection, the relative abundance of each taxon in both racial groups and genital locations were compared employing LEfSe analyses. Table 8 shows that the presence of infection enriched the relative abundance of the taxon *Atopobium* in the vagina of Aboriginal women, and of the taxa *Acinetobacter, Anaerococcus, Gardnerella, Megasphaera*, and *Prevotella* in the vagina of Non-aboriginal women. Notwithstanding that no significant overall differences were found between the placental microbiomes of healthy Aboriginal women and those who experienced an infection, LEfSe analyses showed that in this group of women the presence of infection resulted in enrichment of the relative abundances of *Pseudomonas* and a taxon of the order Sphingomonadales (Table 8). A significant difference was found between the placental microbiomes of healthy Non-aboriginal women and those who experienced an infection during pregnancy and LEfSe analyses showed that infection enriched the taxon *Corynebacterium* (Table 8).

**Table 8:** Taxa detected in higher relative abundances in the presence of infection in Aboriginal or Non-aboriginal women.

**Taxon**	**Racial group**	**Location**	**LDA score**	***p*-value**
*Atopobium*	Aboriginal	Vagina	2.274	
*Pseudomonas*	Aboriginal	Placenta	4.854	0.009
Sphingomonadales	Aboriginal	Placenta	3.897	0.022
*Acinetobacter*	Non-aboriginal	Vagina	2.720	0.016
*Anaerococcus*	Non-aboriginal	Vagina	2.837	0.016
*Gardnerella*	Non-aboriginal	Vagina	2.820	0.011
*Megasphaera*	Non-aboriginal	Vagina	3.103	0.016
*Prevotella*	Non-aboriginal	Vagina	3.029	0.040
*Corynebacterium*	Non-aboriginal	Placenta	2.927	0.016

Focusing on Aboriginal women with infection, Table 9 summarizes microbiome data in the presence of *Pseudomonas* and *E. coli/Shigella* in the genital microbiomes of these women, and clinical data on *Streptococcus* B status, PTB and infant sepsis. *Pseudomonas* was detected in 25% of women in this group, and high rates of PTB and infant sepsis were noticed.

**Table 9:** Clinical and microbiome sequencing data of Aboriginal women who presented signs of infection.

**Woman**	***Pseudomona*s (%)**	**GBS**	***E. coli/Shigella* (%)**	**PTB**	**Sepsis**	**Weight (g)**
A6003	N	?	2; P	N	Y	3,890
A6004	N	Y	6; P	N	Y	3,280
A6005	99; P, V	N	N	Y	Y	2,140
A6006	7; P	Y	1; P	Y	N	2,580
A6007	99; P	?	N	N	Y	2,440
A6009	93; P	?	N	N	N	4,190
A6010	N	Y	22; V	Y	Y	2,840
A6011	N	Y	N	N	N	2,410
A6014	N	Y	12; P	N	N	3,440
A6018	N	Y	6; P	N	N	4,130
A6021	N	N	N	Y	Y	3,170
A6023	N	Y	N	N	N	3,660

*Taxa relative abundances are given as numbers. Genital location: P-placenta, V-vagina. GBS: Streptococcus Group B status; (?) unknown status. PTB: preterm birth. Sepsis: status. Weight at birth in g*.

A number of taxa have been associated with PTB and sepsis. Table 10 shows the relative abundance and the genital location of a dozen of these taxa with abundances > 1% and frequencies > 15% in the microbiomes of Aboriginal women who had signs of infection during pregnancy.

**Table 10:**
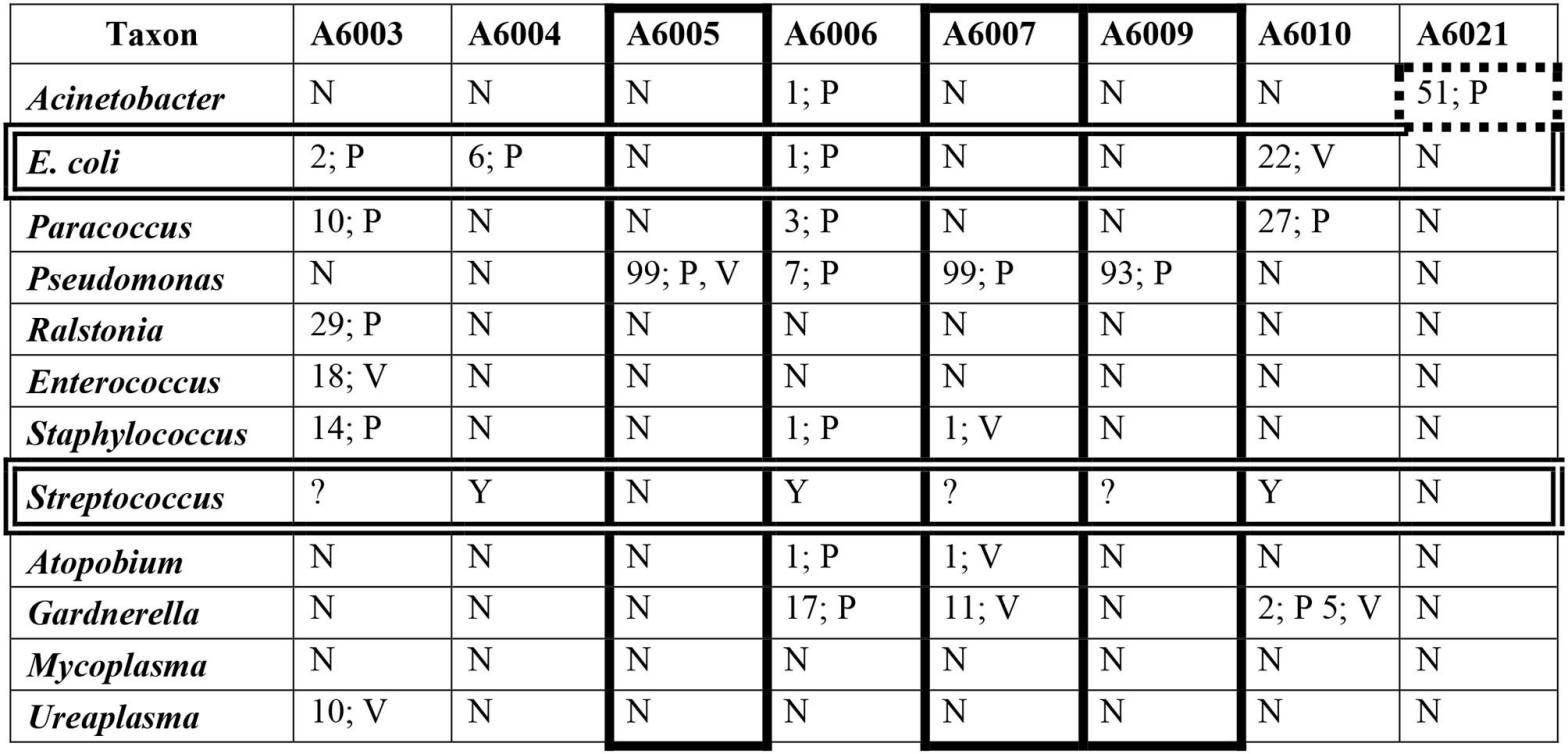
The relative abundances of a dozen taxa in the genital microbiomes of eight Aboriginal women who delivered preterm and/or the infant had sepsis.

Taxa relative abundances are given as percentage numbers. Genital location: P-placenta, V-vagina. Continuous dark borders indicate three women in whose placental microbiome Pseudomonas was the dominant taxon. The double line borders indicate the abundance of E. coli/Shigella and Streptococus Group B. The discontinuous border indicates the abundance of Acinetobacter.

## Discussion

Investigations of the genital microbiomes of pregnant Aboriginal Australians were needed to understand their characteristics and identify taxa whose presence were associated with infection in this population. The study compared the genital microbiomes at the time of delivery of pregnant Aboriginal and Non-aboriginal women living in the Pilbara region employing high throughput 16S-rRNA sequencing. Some differences in demographic and clinical characteristics between both racial groups were statistically significant; however, the small size of each group in the cohort and the values of these differences made them in most cases not relevant to understanding the microbiological data. Nonetheless, the differences in infection and PTB rates between both groups of women helped to interpret the abundances of some taxa (Table 1).

The relative abundances of various Phyla showed differences between vaginal and placental microbiomes and between the microbiomes of Aboriginal and Non-aboriginal women (Figure 1), opening up the possibility for significant differences at lower taxonomic levels. The predominance of Firmicutes in the vagina was in agreement with the results of previous studies including Black, Asian, Caucasian and Hispanic American women (Aagaard et al., 2012; MacIntyre et al., 2015). The predominance of Proteobacteria in the placental microbiome agreed with results for Caucasian European (Collado et al., 2016) and Caucasian and Black American women (Parnell et al., 2017), but not with those of Chinese women in whom the predominant placental Phylum was Firmicutes (Zheng et al., 2015), indicating a dependence of placental bacterial populations on racial background.

The overall composition of the microbiomes of women in both racial groups showed significant differences in distLM calculations. However, comparison of the microbiomes of Aboriginal and Non-aboriginal women yielded similar OTU composition, species richness, evenness and Shannon diversity index for the vaginal and placental microbiomes (Table 2). A prospective study by Hyman et al. (2014), found that the vaginal microbiome species diversity was greatest among African American (*n* = 8) followed by Hispanic (*n* = 13) women compared with that of Asian (*n* = 16) and Caucasian (*n* = 40) women; in addition, statistically significant differences were found between Asian and Caucasian women relative to African American or Hispanic women. This greater diversity in the vaginal microbiome of women of African descent was confirmed in an investigation comprising 300 pregnant women (Serrano et al., 2019). A study of the vaginal microbiome in the third trimester by Fettweis et al. (2019) that included women of African ancestry (71 delivered at term; 35 preterm) and European ancestry (13 delivered at term; 6 preterm) observed greater species diversity in women who delivered preterm. In contrast, Stout et al. (2017) reported that the vaginal microbiome of 24 non-African American women had lower species diversity than that of 53 African American women. It remains to be determined whether the absence of significant differences observed in the present work between Aboriginal and Non-aboriginal women will persist in analyses of larger numbers of samples. The presence of infection resulted in significant changes in evenness and diversity (Table 2) in agreement with the results of a study that observed greater species diversity in women who delivered preterm and included women of African ancestry (*n* = 106) and European ancestry (*n* = 19) (Fettweis et al., 2019).

Comparison of the vaginal and placental microbiomes indicated that they clustered separately in nMDS plots (Figure 2), and distLM calculations showed a significant difference between both genital locations in agreement with the results of previous studies (Aagaard et al., 2014). In healthy pregnant women, midpoint vaginal microbiomes are stable in time and are less rich and diverse than in healthy non-pregnant women (Aagaard et al., 2012; Walther-António et al., 2014). The numbers of OTU, richness and evenness, but not the Shannon diversity, were different between vaginal and placental samples (Table 2). Analyses by location and racial background showed nMDS clustering of the placental microbiomes in these populations (Figure 2); but no significant differences were found in PERMDISP calculations. Nonetheless, the differences in OTU composition, richness and evenness remained after stratification by racial background (Table 2). The results of this work suggested that the microbiome in the normal placenta has significant lower abundance, richness and diversity than in the vagina in agreement with previous investigations (Aagaard et al., 2014; Taddei et al., 2018; Seferovic et al., 2019).

Differences in relative abundances of specific taxa with frequencies > 15% were identified between both genital microbiomes (Table 4). More taxa were enriched in the vagina than in the placenta reflecting the greater diversity and abundances found in the former genital location; exceptions were *Enterococcus* and *Flavobacterium*. The higher relative abundance of *Enterococcus* found in placental samples agreed with the results of a study comparing placental microbiomes of Chinese women who gave birth to normal or low weight infants (Zheng et al., 2015); also, in a study of 1,832 placental samples from Chinese women with healthy pregnancies, *Enterococcus* was recovered by cultivation in 24% of the placental samples second in abundance only to *E. coli*. (Zhou et al., 2018). Viable bacteria have been detected in 15 normal placentas from pregnancies delivered at term by Caesarean section with *Enterobacter* and *Escherichia/Shigella* as the most abundant taxa and smaller abundances of *Lactobacillus, Staphylococcus* and *Streptococus* taxa (Collado et al., 2016). *In situ* hybridization of the placentas of 52 women in term and preterm births without evidence of infection and independent of delivery mode yielded relative abundances greater than 5% for *Lactobacillus, Prevotella, Staphylococcus*, and *Streptococcus* taxa. (Seferovic et al., 2019).

*Flavobacterium* has been found as a common contaminant in reagents, but after applying the quality control with the reagents employed in the sequencing and in the analyses described in the Methods section, this taxon appeared at significant relative abundances in a number of placental microbiomes of both Aboriginal and Non-aboriginal women, and at over twice the abundance in the latter group of women. This higher relative abundance identified in the participating women, concurred with the results of a study of the endometrial microbiome of women undergoing assisted reproductive technology treatments (Franasiak et al., 2016).

The microbiomes of Aboriginal and Non-aboriginal women showed differences in relative abundances of taxa with frequencies > 15% (Table 5). The results were consistent with those of genital microbiome studies of healthy women from various racial backgrounds (Romero et al., 2014; MacIntyre et al., 2015). An unusually high relative abundance of *Pseudomonas* was present in genital microbiomes of Aboriginal women, three times more abundant than in the microbiomes of Non-aboriginal women. *Pseudomonas* has been detected in placental samples of overweight and obese Caucasian Australian women (Gomez-Arango et al., 2017); also, it has been identified at high relative abundance in samples from the endometrium of the healthy control group of a study of Chinese women with endometrial polyps (Fang et al., 2016), endometrial and Fallopian tube samples of a large cohort of reproductive age Chinese women operated for conditions not known to involve infection (Chen et al., 2017), and endometrial samples of women with chronic endometritis (Moreno and Franasiak, 2017). Considering that the distLM logistic regression confirmed that significant differences in microbiomes were due to racial background and genital location (Table 3), these results suggested that colonization with *Pseudomonas* is characteristic of the Aboriginal population of this study.

Comparison of the vaginal microbiomes of Aboriginal and Non-aboriginal women yielded significant differences in the relative abundances of a number of taxa (Table 6). In the vagina of Non-aboriginal women, *Corynebacterium* was found only in negligible or very small concentrations; also, these women had negligible or very low *Lactobacillus* spp. abundance. The highest relative abundances of *Corynebacterium* found in Aboriginal women with no clinical signs of infection suggested that the taxa present were non-pathogenic species. These species of corynebacteria produce organic acids and stimulate the production of anti-inflammatory cytokines that enhance the capacity of the vaginal mucosa to protect against opportunistic and pathogenic microorganisms (Gladysheva and Cherkasov, 2018); *Corynebacterium* may play a similar role in the vaginal microbiome of Aboriginal women.

Vaginal microbiomes abundant in *Atopobium* and low in *Lactobacillus* can reach up to 40% amongst Black and Hispanic women (Ma et al., 2012), and *Atopobium* and *Megasphaera* have been associated with vaginal infections (Hocevar et al., 2019). In the vaginal microbiota of Aboriginal women *Atopobium* was commonly found in microbiomes with low *Lactobacillus* abundance but it was not associated with clinical signs of disease. *Megaspahaera* also was not found in women with clinical signs of infection.

Differences in the community structures (CST) have been identified in the vaginal microbiomes of non-pregnant (Ravel et al., 2011) and of pregnant (Freitas et al., 2018) healthy women from various racial backgrounds. CST III is found at a greater frequency in women with Asian, Black and Hispanic racial backgrounds, and CST I is found in Asian and Caucasian women Ravel et al., 2011; Gajer et al., 2012; Freitas et al., 2018). In terms of population structures, the predominant species in the microbiomes of Aboriginal women was *L. iners* which is associated with vaginal community state type CST III, and in Non-aboriginal women the most abundant taxon was identified as *L. crispatus* which is associated with CST I. The study by Hyman et al. (2014) concluded that *L crispatus* had a higher relative abundance in the vaginal microbiome of Caucasian participants, and *L iners* in African-American and Hispanic participants, and both species had similar relative abundances in Asian women. An investigation of the vaginal microbiome in the third trimester found higher relative abundances of *L. iners* in women of African descent, and of *L. crispatus* in women of European descent (Fettweis et al., 2019). Serrano et al. (2019) studied a matched cohort of women of African ancestry (*n* = 49) and European ancestry (*n* = 41) who delivered at term and found in the third trimester a predominance of *L. iners* in the former group and of *L. crispatus* in the latter. In contrast, Stout et al. (2017) determined that *L. iners* was significantly more abundant than *L. crispatus* in a study comprising both African Americans and non-African Americans. *Gardnerella* and *Atopobium* are dominant genera in the vaginal microbiome of CST IV, and present at lower abundances in the other CST (Freitas et al., 2018). In the study of women with predominantly African ancestry by Fettweis et al. (2019), the next most abundant taxa after *Lactobacillus* were *Gardnerella*, BVAB1, *Atopobium* and *Prevotella* cluster 2. No significant differences between the abundance of *Atopobium* and *Prevotella* were observed for women of African or European ancestry, but *Gardenerella* was more abundant in women of African ancestry. The work of Serrano et al. (2019) showed a linkage between *Gardnerell*a clade 3 and women of African ancestry, and no *Gardnerella* showed a significant association with women European ancestry. On the other hand, Stout et al. (2017) found no difference in *Gardnerella* abundance between were African Americans and non-African Americans. In Aboriginal women the most abundant vaginal taxon was *L. iners* followed by *L. crispatus, Gardnerella, Pseudomonas*, and *Enterococcus*. Considering that the genital microbiomes of these women were dominated by *L. iners*, and the abundance in healthy Aboriginal women of other taxa such as *Corynebacterium, Atopobium* and *Megaspahera* suggested in the context of the reduced diversity of the vaginal microbiomes of pregnant women, that there were some similarities with the vaginal microbiome of women of African descent, but overall the Aboriginal pregnant women showed an uncommon vaginal community structure.

Significant differences between the placental microbiomes of Aboriginal and Non-aboriginal women were the result of LEfSe analyses (Table 7). In addition to *L. crispatus*. The placental microbiomes of Non-aboriginal women were enriched with *Streptococcus* and *Veillonella*. The former taxon has been found commonly in placental samples of women of various racial backgrounds: Chinese (Zheng et al., 2015; Zhou et al., 2018), Caucasian European (Collado et al., 2016), Caucasian and African American (Parnell et al., 2017), Caucasian Australian (Gomez-Arango et al., 2017), and African Malawian women (Doyle et al., 2017), suggesting the colonization of a broad range of racial backgrounds. *Streptococcus* has been identified at high relative abundance also at the endometrium: in samples of Chinese women with endometrium polyps (Fang et al., 2016), of predominantly Caucasian American (Tao et al., 2017) and Caucasian Italian (Moreno and Simon, 2018) women undergoing IVF cycles. Similarly, *Veillonella* has been identified in the placenta of women from various racial backgrounds (Aagaard et al., 2014; Zheng et al., 2015; Doyle et al., 2017; Gomez-Arango et al., 2017; Moreno and Simon, 2019).

In Aboriginal women in addition to *L. iners*, the placental microbiomes were enriched with *Paracoccus* and *Pseudomonas* (Table 7). The former taxon has been identified in the placenta of women from only few racial backgrounds and at low relative abundances (Zheng et al., 2015; Theis et al., 2019), and has been considered a contaminant by others (Perez-Muñoz et al., 2017; Leiby et al., 2018). In the present study after elimination of OTU with an abundance <1% in the negative controls, *Paracoccus* was present at relative abundances > 1% and with a frequency higher than 15%, as were all the other taxa presented in tables.

Comparisons of the effects of infection on the genital microbiomes of Aboriginal women did not yield any overall significant differences; likewise, for the vaginal microbiomes of Non-aboriginal women. However, at the taxon level, LEfSe analyses showed important differences in the presence of infection (Table 8). Potential effects of colonization by *Atopobium, Corynebacterium, Gardnerella*, and *Megasphaera* and have been discussed. The presence of *Acinetobacter* has been associated with PTB and neonatal infections in American women (He et al., 2013), spontaneous preterm and chorioamnionitis in an Australian Caucasian woman (Quinlivan et al., 2014), and vaginal infection in Indian women (Gopalan et al., 2017). Genital infections of *Acinetobacte*r during pregnancy are associated with adverse outcomes in various racial backgrounds.

Post-delivery increases in the abundance of *Anaerococcus* and *Prevotella* with disturbance in the vaginal microbiota unrelated to gestational age at delivery were observed in a population of mostly Caucasian American women (DiGiulio et al., 2015). In contrast, significant *Anaerococus* vaginal colonization was detected in studies with mostly African American with normal pregnancies (Romero et al., 2014) and of Chinese women with uncomplicated pregnancies (Chen et al., 2019). *Anaerococcus* appeared to colonize the female genital tract of women of various racial backgrounds albeit with different pregnancy outcomes. In a study with mostly African American women, *Prevotella* was found with higher abundance in the vagina of women who delivered preterm (Subramaniam et al., 2016). Several *Prevotella* spp. were associated with both term and preterm in predominantly Caucasian American women. *Prevotella amnii* and *P. tannerae* had greater prevalence in the term cohort, whereas *P. timonensis, P. bivia, P. corpori*s, and *P. bucalis* were more prevalent in the preterm group (Freitas et al., 2018). *Prevoltella* was strongly associated with vaginal infection in a study of mostly Caucasian Brazilian women (Dobbler et al., 2019). These results suggest an overall association of *Prevotella* with vaginal infection in different racial backgrounds.

Amongst the 23 Aboriginal women in the study, 12 presented clinical signs of infection. The relative abundance of *Pseudomonas, E. coli/Shigella* and the prevalence of GBS in Aboriginal women with sign of infection is given in Table 9; included in the table are the metadata for PTB and sepsis. In the placental microbiome of 3 of these women the dominant taxon was *Pseudomonas* with relative abundances > 93%; this taxon was dominant also in the vaginal microbiome (99% relative abundance) of one of these women. *Pseudomonas* was identified at low relative abundance in another Aboriginal woman. This taxon has been found in the placenta of women from different racial backgrounds, e.g., Chinese women who delivered of low birth weight infants (Zheng et al., 2015), Chinese women with gestational diabetes mellitus (Zheng et al., 2017), overweight and obese Caucasian Australian women (Gomez-Arango et al., 2017), and African American women who delivered at term (Theis et al., 2019). Commonly, intrauterine presence of *Pseudomonas* has been associated with infection but not in all instances.

Many taxa have been associated with infection during pregnancy (DiGiulio et al., 2015; Freitas et al., 2018). Table 10 shows the relative abundance of 12 of these taxa in eight of the Aboriginal women who had adverse pregnancy outcomes, either PTB and/or neonatal sepsis. In women A6005, A6007, and A6009 there is a correlation between these outcomes and the presence of *Pseudomonas* at high relative abundance in the absence of significant abundance of other potential pathogens. The presence of several potential pathogens at low and intermediate abundances in the genital microbiomes of woman A6003 would account for her infection. The Group B *Streptococcus* positive status of women A6004, A6006, and A6010 may explain the presence of infection in these participants. In addition, the low abundance of *Pseudomonas, Gardnerella* and other potentially pathogenic taxa in woman A6006, and the intermediate abundance of *E. coli* and *Paracoccus* in woman A6010 may have contributed to their respective infections. Participant A6021 did not show abundance of *E. coli*, GBS or other taxa that would explain satisfactorily that she delivered preterm a child with sepsis. However, *Acinetobacter* has been associated with preterm birth and was detected at relatively high abundance in the placenta of this woman.

Early-onset neonatal sepsis almost always is associated with pathogenic microorganisms acquired from the mother during pregnancy or at birth (Klinger et al., 2009); they are commonly GBS and *E. coli* (Schrag et al., 2016), but a significant proportion of EOS is due to other infections. *Pseudomonas* has been associated with PTB and late-onset sepsis (Harnaen et al., 2015), but in Australia and New Zealand also with EOS (Braye et al., 2019; Singh et al., 2019).

The abundance of *Pseudomonas* and *E. coli*, and the GBS status in the genital microbiomes of Aboriginal and Non-aboriginal women in this study are given in Supplementary Table 2, together with clinical data on PTB and sepsis. These data suggested the association of *Pseudomonas* with PTB and/or sepsis, particularly in Aboriginal women, also in the absence of other potential infections.

## Conclusion

The study investigated the genital microbiomes at delivery in Aboriginal women of the Pilbara region of Western Australia by comparing them to Non-aboriginal women in the same region. The study is the first report on the genital microbiomes of pregnant Australian Aboriginal women using culture independent techniques. The results support previous findings that the most abundant vaginal Phyla during pregnancy are Firmicutes, Proteobacteria, and Actinobacteria, and that the vaginal microbiome of normal pregnancy commonly consolidates into communities dominated by *Lactobacillus*. At a global level distLM analyses yielded differences between the genital microbiomes of both groups of women, and at the taxon level significant differences were found between the vaginal or placental microbiomes of Aboriginal and Non-aboriginal women. *Pseudomonas* appeared to be associated with infection and adverse pregnancy outcomes, particularly in the group of Aboriginal women.

The study focused on a rural population with a high rate of spontaneous preterm birth and demonstrated differences in the microbiomes at the taxon level in women with infection. The findings served to enhance the current understanding of microbiota associated with health and disease in Aboriginal and Non-Aboriginal women, and will help the development of targeted prevention of chorioamnionitis. Further studies with larger cohorts will serve to verify the results of this work and the routes of invasion of pathogenic bacteria into the intra-uterine environment that would enable earlier detection of subclinical infection, and allow for further reduction of premature deliveries for both Aboriginal and Non-aboriginal women.

## Data Availability Statement

The datasets generated for this study can be found in the European Nucleotide Archive under the accession number PRJEB39698.

## Ethics Statement

The studies involving human participants were reviewed and approved by WA Country Health Service Human Research Ethics Committee (HREC #2015/40) and Western Australian Aboriginal Health Ethics Committee (HREC #686). The patients/participants provided their written informed consent to participate in this study.

## Author Contributions

ND, JQ, and GM contributed to the design of the study. ND and JQ acquired the data. ND, NC-R, and GM helped with the analyses and interpretation of the data. ND, JQ, NC-R, and GM wrote the manuscript. All authors contributed to the article and approved the submitted version.

## Conflict of Interest

The authors declare that the research was conducted in the absence of any commercial or financial relationships that could be construed as a potential conflict of interest.
