# Supplementary material for: Comparison of the Genital Microbiomes of Pregnant Aboriginal and Non-aboriginal Women
Source: Front Cell Infect Microbiol. 2020 Oct 29;10:523764. doi: 10.3389/fcimb.2020.523764 (PMC7658313; doi:10.3389/fcimb.2020.523764)
Supplement: Supplementary file 1 [file Data_Sheet_1.docx]

**Supplementary material**

**Table S1. Relative abundances of four taxa in the vagina and the placenta in Aboriginal and Non-aboriginal mothers who gave birth through the vagina.** The four taxa were found to be at greater abundances in the vaginal microbiomes relative the placental microbiomes in some mothers and vice versa in other mothers. The data show significant differences between the abundances in both genital locations.

| *Taxon* | Aboriginal Mothers  Abundance (%) | | | Non-aboriginal Mothers  Abundance (%) | | |
| --- | --- | --- | --- | --- | --- | --- |
|  | Participant | Vagina | Placenta | Participant | Vagina | Placenta |
| *Atopobium* | A6018 | 8.6 | 0.0 | N6017 | 8.1 | 0.1 |
|  | A6014 | 0.2 | 9.0 | N6018 | 2.5 | 14.3 |
| *Gardnerella* | A6022 | 5.8 | < 0.1 | N6020 | 8.5 | 0.0 |
|  | A6020 | 0.0 | 11.5 | N6014 | 0.0 | 2.7 |
| *Prevotella* | A6001 | 12.5 | 0.0 | N6027 | 1.7 | <0.1 |
|  | A6019 | 0.1 | 28.3 | N6002 | <0.1 | 9.9 |
| *Staphylococcus* | A6023 | 10.5 | <0.1 | N6016 | 2.1 | 0.0 |
|  | A6012 | 0.1 | 3.7 | N6003 | 0.0 | 6.6 |

**Table S2. Clinical and microbiome sequencing data of women with *Pseudomonas* infection.**

A: Aboriginal woman. N: Non-aboriginal woman. Taxa relative abundances are given as percentage numbers. Genital location: P-placenta, V-vagina. Status: Yes: Y; N: No; Unknown: ?.

| **Woman** | ***Pseudomonas*** | ***Streptococcus* B** | ***E. coli*** | **Birth mode** | **PTB** | **Sepsis** |
| --- | --- | --- | --- | --- | --- | --- |
| **A6005** | 99; P, V | N | N | Vaginal | Y | Y |
| **A6006** | 7; P | Y | 1; P | Vaginal | Y | N |
| **A6007** | 99; P | ? | N | Vaginal | N | Y |
| **A6009** | 93; P | ? | N | Caesarean | N | N |
| **N6005** | 97; P | ? | N | Caesarean | N | N |
| **N6006** | 41; P | Y | N | Vaginal | Y | N |
| **N6008** | 93; V | ? | 12; P | Vaginal | N | N |
